# Liposomes for Topical Use: A Physico-Chemical Comparison of Vesicles Prepared from Egg or Soy Lecithin

**DOI:** 10.3797/scipharm.1305-11

**Published:** 2013-07-14

**Authors:** Lívia Budai, Nóra Kaszás, Pál Gróf, Katalin Lenti, Katayoon Maghami, István Antal, Imre Klebovich, Ilona Petrikovics, Marianna Budai

**Affiliations:** 1 Semmelweis University, Department of Pharmaceutics, Hőgyes E. u. 7., H-1092, Budapest, Hungary.; 2 Semmelweis University, Department of Biophysics and Radiation Biology, Tűzoltó u. 37-47., H-1094, Budapest, Hungary.; 3 Semmelweis University, Faculty of Health Sciences, Department of Morphology and Physiology, Vas u. 17, H-1088, Budapest, Hungary.; 4 Sam Houston State University, Department of Chemistry, TX-77340, Huntsville, USA.

**Keywords:** Natural lipid, Multilamellar vesicle, Macroscopic/microscopic Appearance, Topical drug delivery

## Abstract

Developments in nanotechnology and in the formulation of liposomal systems provide the opportunity for cosmetic dermatology to design novel delivery systems. Determination of their physico-chemical parameters has importance when developing a nano-delivery system. The present study highlights some technological aspects/characteristics of liposomes formulated from egg or soy lecithins for topical use. Alterations in the pH, viscosity, surface tension, and microscopic/macroscopic appearance of these vesicular systems were investigated. The chemical composition of the two types of lecithin was checked by mass spectrometry. Caffeine, as a model molecule, was encapsulated into multilamellar vesicles prepared from the two types of lecithin: then zeta potential, membrane fluidity, and encapsulation efficiency were compared. According to our observations, samples prepared from the two lecithins altered the pH in opposite directions: egg lecithin increased it while soy lecithin decreased it with increased lipid concentration. Our EPR spectroscopic results showed that the binding of caffeine did not change the membrane fluidity in the temperature range of possible topical use (measured between 2 and 50 °C). Combining our results on encapsulation efficiency for caffeine (about 30% for both lecithins) with those on membrane fluidity data, we concluded that the interaction of caffeine with the liposomal membrane does not change the rotational motion of the lipid molecules close to the head group region. In conclusion, topical use of egg lecithin for liposomal formulations can be preferred if there are no differences in the physico-chemical properties due to the encapsulated drugs, because the physiological effects of egg lecithin vesicles on skin are significantly better than that of soy lecithin liposomes.

## Introduction

Developments in nanotechnology and in the formulation of liposomal systems provide the opportunity for cosmetic dermatology to design novel delivery systems. Beyond the efficacy and safety of nanomaterials, the determination of their physico-chemical parameters also has importance when developing a new nano-delivery system [1–4]. Nanomaterials – specifically liposomes – have the potential to make radical changes in the form of drugs and cosmetic delivery [5]. Although liposomes were first described by Bangham in 60’s, the first therapeutic skin agent – an anti-mycotic agent, econazole – encapsulated within lipid vesicles was commercialized shortly before 1990 [1, 6, 7].

The application of liposomes in skin treatment is based on the convenience that the spherical vesicles may encapsulate a wide range of active ingredients. Furthermore, the bilayer structure of lipid vesicles has similarity to that of cellular membranes. As a result of this similarity, the liposomes are able to interact with the cutaneous cells in different ways, such as endocytosis, fusion, or exchange of lipids. The latter has a specific impact in topical preparations and dermocosmetics, since it may alter the physical properties (*e.g.* elasticity) of the skin [1].

Liposomes are able to deliver active ingredients in a concentration-dependent manner across the stratum corneum, the main barrier of the skin. Multilamellar vesicles (MLV) can deliver active ingredients within 30 min to the stratum corneum, epidermis, and dermis in significantly higher concentrations than conventional topical preparations *e.g.* creams, lotions, gels, or emulsions [8]. In addition, liposomes provide valuable raw materials for the regeneration of skin by replenishing lipids and moisture. Even in the absence of humidity, liposomes can improve the skin’s elasticity and barrier function, which are considered to be among the main causes of skin aging [8].

Lecithins of a different origin are one of the most commonly used phospholipids in biological studies. Depending on the origin, lecithins differ in *i.)* composition, including fatty acid chains’ lengths and degree of unsaturation; *ii.)* in polar head groups whose most frequent form is the choline group. Due to these differences, the physico-chemical properties of lecithins may vary depending on the origin.

Literature references [9–12] refer to a number of aspects of the chemical stability of liposomes prepared from phospholipids of a different origin. Despite the difference in the lipid composition, no appreciable differences were observed in the loading capacities when calcein was encapsulated within liposomes prepared from egg lecithin or the less expensive vegetable or soy lecithin [10]. In contrast, lot of literature data suggest that the chemical composition of lipids can impact the physico-chemical properties of liposomes, their interaction with ingredients, and consequently their *in vivo* fate. Some of the observations briefly summarized above raise the importance of the knowledge of physico-chemical parameters which can influence the migration and diffusion of molecules in the delivery matrix, thus the release and skin penetration. In this respect, liposome formulations prepared with egg phosphatidylcholine showed significantly higher hydration effects on human skin compared to liposome formulations prepared with soy lecithin [8].

Although it has been studied for a very long time, caffeine still seems to be a drug of current interest regarding its interaction with biologically small- or macromolecules, and macromolecular systems. Very recent articles addressed the protonation, H-bonding ability [13, 14], or dimerization connected with the solvation of the caffeine [15], emphasizing that in some cases, hydrophobic interactions can lead to the binding of caffeine *e.g.* to hemocyanin [16]. From a pharmaceutical point of view, differences were observed in the release of the caffeine-loaded gel systems depending not only on the concentration of caffeine, but also on the concentration of the gel-forming material. During the skin permeation study, the flux of the active ingredient was more significant in the case of a lower concentration of the gel-forming polymer [17]. In a recent work on phospholipid bilayers interacting with caffeine, changes in the membranes’ electrical resistance were observed, but were not in the liposomal permeability or in the DSC-characteristic [18]. This can suggest specific interactions between caffeine and the phospholipid molecules. As caffeine possesses a relatively small partition coefficient between the water and oil phases [19], the detection of fluidity changes at different levels of the lipid moiety could be feasible using electron spin resonance spectroscopy.

The present study is aimed at *i.)* investigating the concentration-dependent effect of lecithin-liposomes on the pH, viscosity, and surface tension of the vesicular systems prepared from egg (EL) or soy lecithin (SL); *ii.)* determining and comparing the encapsulation efficiency and zeta potential, when the model substance caffeine was encapsulated within the two types of lecithins; *iii.)* characterizing the membrane fluidity changes due to caffeine encapsulation by measuring the electron paramagnetic resonance (EPR) spectra of spin probes incorporated in the liposomal bilayer.

## Results and Discussion

### Comparison of Selected Physico-Chemical Properties

**Fig. 1:**
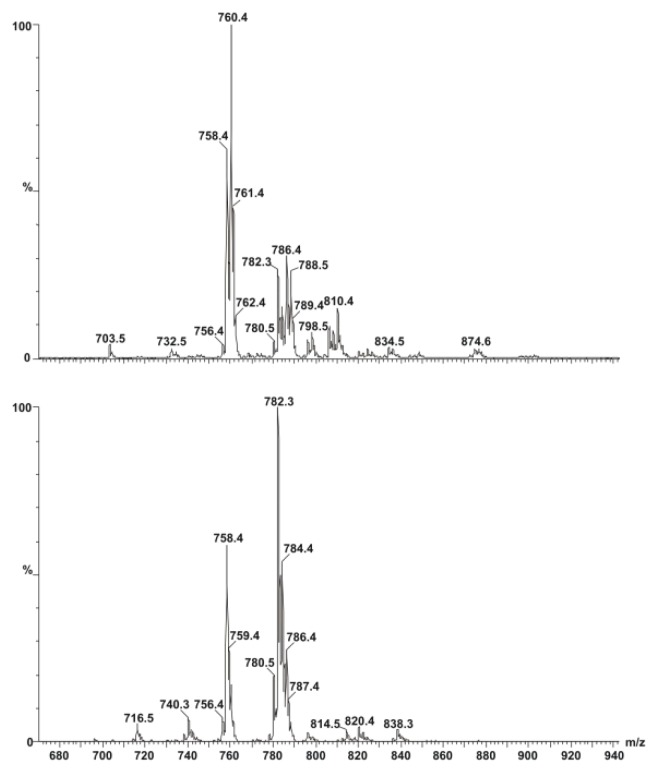
Positive mode mass spectra of egg- (above) and soy- (below) lecithin. Lipid samples (dissolved in methanol) were injected with a flow speed of 0.1 ml/min. Capillary voltage was 4000 V and fragmentor was 70 V.

*Mass spectrometry* was applied to check the actual differences in the compositions of the two lecithin samples, as due to the natural origin of lecithins used in our experiments, the ratio of saturated to unsaturated fatty acid chains may differ. Figure 1 shows MS intensities in percentage values relative to the maximal intensity. In addition, for quantifying our MS measurements, the spectral intensities were normalized for the sum of the intensities measured in the spectral range of m/z values between 700 and 900 and the calculated relative contributions are summarized in Table 1. Some characteristic m/z values can be identified in both types of lecithin, however, the spectra also possess remarkable differences. A more detailed assignment can be made using known MS spectra of the lipid mixtures, comparing regions of the spectra coming from lipids having different chain-length and head groups [24–26]. Inspection of the MS spectra at the region between 650 and 735 m/z values suggests that one can exclude the presence of phosphatidyl-ethanolamine, phosphatidyl-glycerol derivatives possessing lipid chains of 16–20 carbon atom lengths. The same is valid for DPPC, having an m/z value of 734. According to the literature data, the m/z value of 758.4 belongs to a phosphatidylcholine head group possessing lipid with fatty acid chains of 16 and 18 carbon atoms, with two double bonds altogether. In both types of lecithins, this region contributed to the whole signal with almost the same values: 13% and 12% for soybean and egg lecithins, respectively. However, by taking the contributions in the region from 756 to 762 m/z values, remarkable differences can be observed: egg lecithin gave 50%, while soybean lecithin possessed only 27%. Moreover, contributions in this interval due to POPC were found to be 20% and 3% for egg and soybean lecithins, respectively. In the next region between m/z values of 780 and 790, lipid contributions sum up to 31% and 68% for egg and soybean lecithins, respectively. In this region the m/z value of 782.3 can indicate the presence of lipid molecules with two fatty acid chains possessing 18-18 C-atoms and four double bonds altogether, having merely 5% contribution in egg lecithin, while giving about 23% for soybean lecithin. The presence of an increased number of double bonds may lead to higher membrane fluidity, which we present later on among the EPR measurements. By taking contributions in the range between 780 and 810 m/z values, further signals can be seen with the characteristic m/z value differences of 2, 4, or 6, indicating the alternating numbers of double bonds in fatty acid chains connected either to choline or serine head groups. The more negative zeta potential values measured by us for SL- than for EL-liposomes argue in favour of a higher serine content in the case of liposomes prepared from SL than from EL. In summary, our verification of EL- and SL-composition showed that *i.)* egg yolk lecithin contains less unsaturated lipid than the soybean one; *ii.)* these results combined with those of zeta potential measurements suggest the presence of serine-derivatives.

The ionic charges present on the liposomal membrane deeply influence the permeability, electrical resistance, and the inter-liposomal interactions [18, 27, 28]. The pH-measure-ments on liposomes suspended in deionized, ultrafiltered water have been undertaken to compare the possible differences between SL- and EL-liposomes. Evaluating the effect of the presence of liposomes on the pH of the preparations, we found that in the case of MLVs prepared from soy lecithin, a pH value of 7.2 was measured, while EL showed a pH of 4.0 at a 10 mg/ml concentration. As expected for both types of lecithins, decreasing concentrations of MLVs resulted in smaller deviations from the pH of the deionized ultrapure water than at higher MLV concentrations. As for dermatologic and cosmetic use, the pH of 5.5 is ideal, these liposomal samples – without the addition of buffering auxiliary materials meet this criterion – in a relatively low lipid concentration (~ 0.5 mg/ml) (Table 2.). Thus, at higher lipid concentrations for reaching an optimal pH for topical liposomal preparations instead of distilled water, the use of buffer systems is recommended.

After measuring the viscosity of the liposomal samples, we concluded that for both types of lecithin liposomes, the higher lipid concentrations led to higher viscosity values (Table 2.). The type of lecithin does not influence the viscosity measured at a given lipid concentration. Plotting the viscosity data vs. the lipid concentration resulted in a straight line with a regression coefficient of 0.993 at concentrations between 0 and 10 mg/ml. For 12 and 15 mg/ml lipid concentrations, the viscosity values were lower than expected. The soy or egg origin of the lipids does not seem to have an influence on the viscosity of the samples. On the basis of the above statements, it is clear that the rheological properties (viscosity – lipid concentration function) of the liposomes are different from that of the gel-forming polymers, where higher gel-forming material concentrations lead to exponentially higher viscosity values.

It is well-known that amphiphilic molecules like lipids act as surface-active agents. In agreement with this property, our surface tension measurements on SL and EL liposomes showed (Table 2.) that at 15 mg/ml lipid concentration, the surface tension of the SL and EL samples is only 39.6 and 39.5 mN/m, respectively, while that determined for distilled water is 73.0 mN/m (according to literature data [29] the surface tension of water is 72.88 mN/m at 20 °C). For the aspect of precise dosage, the size of the drops is an essential parameter. Since the presence of liposomes results in the formation of smaller drops, the application of a liposomal preparation requires a larger number of drops, unlike with the liposome-free dosage formulations. The latter two physico-chemical properties may influence the spray angle of a liposomal formulation. For we determined the spray angle of liposomes prepared from both types of lecithins. Our experimental results showed that the presence of liposomes decreased the spray angle. While for the liposome-free system, an average spray angle of 41.4° was measured, for liposomal samples with lipid concentrations from 5 to 15 mg/ml, the spray angles varied between 31.3° and 40.6°. Additionally, in each case they were smaller than those measured for the liposome-free control system. A correlation analysis resulted in a p-value of 0.164 between the lipid concentrations and the spray angles which would suggest no significant correlation between these two quantities. Moreover, no significant difference was observed between the spray angle values measured at the same concentrations of the SL and EL samples. In general, however, higher lipid concentrations resulted in higher spray angles. This suggests that increasing the concentration of liposomes enhances the viscosity to a lesser extent than they reduce surface tension. As the correlation coefficient measures the linear relationship between two parameters, the p-value given above can reflect our supposition that the relationship between the spray angle and liposomal concentration might not be a linear one.

### Zeta Potential and Encapsulation Efficiency for Caffeine-Loaded Liposomes

In general, the partition coefficient, logP_o/w_, is used to characterize the distribution of drugs between lipophilic and aqueous phases. There is also, however, general agreement that the simple use of the logP values for liposomal suspensions can result in deviations from the expected partition due to molecular interactions that can impact the phenomenon [27, 30]. In the case of caffeine, hydrophobic, but also dipol-dipol interactions [13], as well as water penetration into the bilayer membrane, can impact the partition of caffeine between the aqueous and lipid phases. Literature values can be found for logP_o/w_ ranging between ~−0.07–0.5 (thus ~0.85 < P < ~3). We addressed the question if we could detect any change in zeta potential due to the incorporation of caffeine into the liposomes. First we determined the encapsulation efficiency of EL or SL MLVs for caffeine, and found that the measured values do not differ significantly: 31.2 ± 4.4% and 29.2 ± 3.1% for EL and SL, respectively. Similarly, Memmoli et al. found no difference between the two types of lecithins when calcein was incorporated [9–11] in the liposomes of EL or SL.

The measured encapsulation efficiency seemed to be sufficient to detect possible changes in the zeta potentials, because in a previous work on ciprofloxacin-liposomal interaction, we were able to detect alterations of the zeta potential even at as high as 15% encapsulation efficiency [28]. Zeta potentials of SUVs prepared at pH 5.6 from EL and SL were −33.12 ± 1.5 and −47.57 ± 0.83 mV, respectively. The measured zeta potential values combined with the MS results showed that phosphatidylserines are involved in both lecithins. The pK_a_ value of the glycerol-phosphate head group is around 2.6 in the case of serine-phospholipids resulting in a complete dissociation at pH 5.6. On the contrary, the pK_a_ value for the serine’s carboxylic group is around 5.5, leading to about 50% dissociation (expressed as relative charge −0.5). Dissociation of the ammonium group can be negligible due to its pKa value of ~11.5. Since the absolute values of the measured zeta potentials are above the theoretically appointed 30 mV limit required for stability, we can conclude that the use of both lecithins are acceptable for the preparation of stable liposomal formulations. The presence of caffeine in increasing concentrations, however, did not change the zeta potential of either SL or EL liposomes. Although it is known that at pH 5.6 caffeine molecules are uncharged – as the conjugate acid it has a pK_a_ value of about 0.6 – interactions such as dipol-dipol or hydrogen-bonds might electrically shield or alter the surface charge density in the shear plane around the liposomes where the zeta potential is measured. According to these results and considering the hydrophilic feature of caffeine, it can be concluded that the adsorption of drug molecules on the surface of SUVs does not modify the surface charge densities, i.e. the dissociation of the lipid molecules. Recently, a similar observation was obtained also on the surfactant vesicles whose zeta potential did not change after the addition of caffeine [31].

### EPR Spectroscopy

EPR spectroscopy provides information about the molecular rotational motion in the time regime of 10^−4^–10^−12^ s, characterized by the parameter, called the rotational correlation time. Due to the spatial arrangement of the liposomal bilayer, another parameter, the order parameter, is also frequently used to characterize the membrane fluidity. In the first step of spectral evaluations, the use of a simpler, directly observable spectral parameter, the maximal hyperfine splitting constant, 2A_max_, is the preferred characteristic. According to the theoretical considerations and experimental observations, this parameter corresponds to the difference in magnetic field values that can be measured between the so-called low-field maximum and the high-field minimum. An increase in this parameter shows a decreased fluidity that can be connected to a more ordered, more rigid state of the lipid molecules and/or a slower molecular rotational motion. As caffeine possesses a low logP_o/w_ value, its interaction with liposomes is expected to occur close to the head group region. By probing this region, we have chosen two types of nitroxide radicals that can monitor molecular rotational motion close/near the lipid–aqueous interface. One of them, DOX-5, is located at the fifth position of the fatty acid chains, while the nitroxide moiety of the other spin label, HXD, is situated at the head group level. We measured the EPR spectra of DOX-5-labeled MLVs and in line with literature data, we found that the maximal hyperfine splitting values possess remarkable differences for liposomes prepared from EL or from SL in the whole temperature range (between 2 °C and 50 °C) that we studied. At each temperature, higher fluidities have been detected for soybean lecithin than for egg yolk lecithin MLVs. The difference of 2A_max_ was 1.58 ± 0.094 gauss. These results are in accordance with our MS measurements, which demonstrated that SL contains more unsaturated fatty acids than EL, which can lead to higher fluidity. On the contrary, no significant difference in fluidity has been detected between the control and caffeine-containing MLV samples; the average differences of 2A_max_ values for caffeine-treated and untreated samples were 0.03 and 0.02 gauss for SL and EL samples, respectively. This observation suggested that the interaction of caffeine with liposomes observed in our encapsulation experiments did not result in a change of the membrane fluidity that would impact the molecular motion at the fifth carbon atom depth of the bilayer. In other series of experiments, we applied a spin label derivative HXD, which tests the lipid bilayer at the head group level [32]. To draw the maximally possible information, the experimental spectra were fitted by a non-linear least square procedure developed by the Freed’s group [33, 34] which was implemented by us for Windows PCs [22]. We compared three main parameters:

1. average rotational correlation time;
2. ordering potential;
3. order parameter (Table 3).

**Fig. 2:**
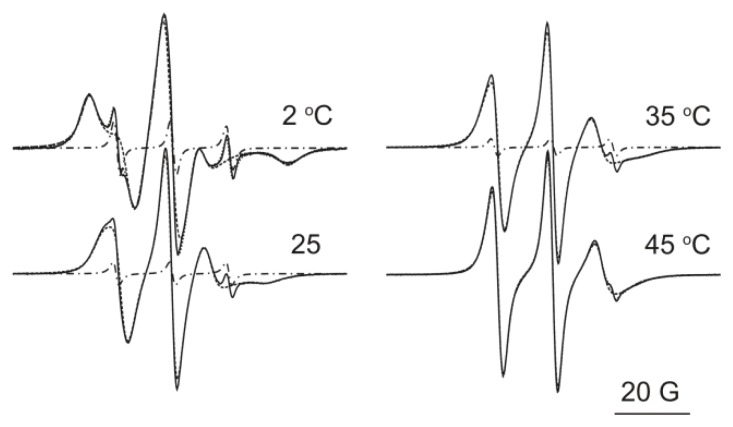
Representative EPR spectra and the fitted components at four temperatures. The solid line represents the experimental spectrum, whereas the long dashed and dot-dashed lines show the components of longer and shorter correlation time, respectively. At 2 °C and 45 °C the sum of the two components are shown in the figure (dotted line).

Figure 2 shows the spectra measured on liposomes prepared from egg yolk lecithin in the presence of caffeine at four selected temperatures. Each spectrum is a superposition of a more mobile and a less mobile component. The less mobile component contributes to the whole spectrum no more than about 3%, determined on the basis of our spectral simulations. Our calculation showed that there is no significant difference between control and caffeine-treated samples either in ordering potentials, in order parameter, or in average correlation time within the limit of error of spectral simulation. These errors were estimated as to give average SEM values for c_20_: ± 0.06, for S: ± 0.02. For the average rotational correlation time, a relative SEM value is the best characteristic, which is ten percentages of the best-fit parameter value. EPR measurements with both types of spin labels suggest that there is no detectable difference in rotational motion either close to the head group at the fifth carbon atom depth or at the bilayer water interface at the head group level.

These observations showed that even if the caffeine binds to the liposomal membrane with as high as about 30% encapsulation efficiency, this binding does not modify either the zeta potential or the rotational motions of the lipid molecules close to the head group region. On the basis of the encapsulation, zeta potential, and EPR measurements, however, we can accept that although the caffeine–membrane interaction seems to be not strong enough to modify the electrostatic interactions and the local rotational correlation time, the binding of the caffeine to the liposomal membrane does occur. From an EPR spectroscopic point of view, the use of spin-labeled caffeine or a similar nitroxide structure could help to directly detect the binding of the caffeine to the liposomal membrane.

## Conclusion

On the basis of the present results, it can be concluded that the origin of lecithin (egg yolk or soybean) does not have a significant impact on the viscosity, surface tension, and spray angle of liposomal preparations with various lipid concentrations. For the model substance, caffeine, the encapsulation efficiency values were not significantly different when it was encapsulated within EL vs. SL MLVs. However, as a consequence of different chemical compositions, differences can be observed in the pH-values of liposomal suspensions prepared from these two types of lipids. Lack of changes in zeta potential and rotational motion due to the interaction of liposomes with caffeine, combined with the measured encapsulation efficiency, might suggest that the encapsulation of caffeine could be the consequence of a solvation sheet closely attached at the aqueous interface of the bilayer surfaces; but this hypothesis requires further verification by other more feasible, sensitive methods.

Concerning liposomal formulations, the topical use of egg lecithin can be preferred, if there are no differences in the physico-chemical properties due to encapsulated drugs, because the physiological effects of egg lecithin vesicles on the skin are significantly better than that of soy lecithin liposomes.

## Experimental

### Materials

L-alpha-phosphatidylcholine from soybean (SL), absolute ethanol, caffeine, and spin label 5-doxyl-stearic acid (DOX-5) were purchased from Sigma Chem. Co., Budapest, Hungary. The nitroxide spin label derivative 4-(*N*,*N*-dimethyl-*N*-hexadecyl)ammonium-2,2′,6,6′-tetramethylpiperidine-1-oxyl iodide (HXD) was from Molecular Probes Inc., Eugene, OR, USA. Egg lecithin (EL) was from Fluka AG, Buchs, Switzerland. Polycarbonate filters with a pore size of 100 and 50 nm used for extrusion were from Schleicher&Schuell GmbH. Dassel, Germany. Rhodamine DHPE (Rhodamine B) was from Invitrogen Corporation USA, CA. Microcon YM-10 centrifugal filter devices with a cut-off value of 10 kDa were purchased from Millipore Inc., Budapest, Hungary. Deionized ultrafiltered water was produced by the Milli-Q system (Millipore Inc., Budapest, Hungary).

## Methods

### Preparation of Liposomes

Multilamellar vesicles from SL or EL were prepared using the thin-film hydration technique. From a stock solution of lipid (usually 20 mg/ml lipid in absolute ethanol), the appropriate volumes were taken and the solvent was evaporated under nitrogen or argon stream and held thereafter in a vacuum-desiccator for 24 hours. The hydration of the lipid films was carried out at about 40 °C, above the main phase transition temperature of lecithins with distilled water. The final concentrations of lecithin were 0.5; 2; 5; 7; 10; 12; and 15 mg/ml. In the case of caffeine-containing MLV-samples, the lipid films were hydrated with an aqueous solution of caffeine (caffeine concentration was 0.3 mg/ml with a lipid concentration of 10 mg/ml). Thus, the molar ratio of caffeine-to-lipid was ~ 1 to 10.

Small unilamellar vesicles (SUV) were prepared from MLVs – 10 mg/ml lipid concentration, hydrated in 10 mM PBS buffer solution (pH 5.6) – by extrusion. Extrusion of liposomes was carried out at 40 °C with 21 and 41 extrusion cycles through polycarbonate membrane filters with pore sizes of 100 and 50 nm, respectively.

In preparing the MLV samples used for EPR spectroscopy, spin label and caffeine were co-solved with lecithin (caffeine-to-lipid molar ratio was 1:5) in a mixture of organic solvent (chloroform:methanol 9:1). The concentrations of the spin labels were 1 and 0.75 mol% for DOX-5 and HXD, respectively.

### Mass Spectrometry

The PE Sciex API 2000 mass spectrometer was used to perform the MS measurements in positive mode. Lipid samples (dissolved in methanol) were injected with a flow speed of 0.1 ml/min. Capillary voltage was 4000 V and the fragmentor was 70 V. A quadrupole scanned over the range m/z 100–1000. A nebulizer and drying gas nitrogen were applied with 35 psi and 13 l/min, respectively.

### pH Measurement

The pH of the liposomal samples was determined by the Hanna Instruments pH 210 Microprocessor pH Meter (Hanna Instruments Service Ltd, Szeged, Hungary). Before each series of measurements, the pH meter was carefully calibrated with two pH-standards in the corresponding pH range, so that the difference between the calibration standards differed only by two pH units. The calibration procedure was repeated if the measurement period was longer than 30 minutes.

### Viscometry

The viscosity of the non-liposomal and liposomal samples with various lipid concentrations was determined at 25 °C by the Vibro Viscometer (SV-type) (Malvern Instruments Ltd., UK) that allowed a sensitive determination of the viscosity within the range of 0.3–10 000 mPa·s with an accuracy of 1% relative error.

### Surface Tension Measurement

The surface tension of the liposomal preparations was determined at 25 °C by stalagmometry. The exact number of drops formed from 2.0 ml sample volumes was used as a measure of the surface tension of a fluid. Surface tension can be obtained by using the well-known relation:

$$\sigma =\sigma_{{\text{H}}_{2}\text{O}}\cdot \frac{\rho }{\rho_{{\text{H}}_{2}\text{O}}}\cdot \frac{{\text{n}}_{{\text{H}}_{2}\text{O}}}{\text{n}}$$

Where σ, σ_H2O_, ρ, ρ_H2O_, n and n_H2O_ are the surface tensions, densities, and drop counts for water and liposomal systems.

### Determination of Spray Angle

The spray angle was determined using a sample holder spray positioned in L=50 mm distance from a vertical silica-covered glass. Methylenblue-colored MLV samples were used to make the sample spots visible. The diameter (d; in mm) of the spots were measured and used for the calculation of the spray angle (α). Spray angle was calculated according to the simple geometrical relation as follows: tg(α/2) = d/(2L).

### Determination of Encapsulation Efficiency for Caffeine

400 μl of freshly prepared caffeine-containing MLV samples were centrifuged with an Eppendorf centrifuge (11000 × g; 20 min) through Centrifugal Filter Devices with a cut-off value of 10 kDa. The concentration of caffeine in the filtrate, representing the amount of caffeine in the aqueous phase, was determined by spectrophotometry (Unicam 2 UV/VIS spectrophotometer) at a wavelength of 273.6 nm. The absorbance of the aqueous caffeine solution used in the hydration procedure (0.3 mg/ml) served as 100% for the determination of encapsulation efficiency.

### Zeta Potential Measurements

Zeta potential measurements were carried out at 25 °C by the Zetasizer Nano ZS (Malvern Instruments Ltd., Worcestershire, UK) on SUVs made of egg and soy lecithin. 100 μl of SUV with 10 mg/ml lecithin concentration was diluted with an appropriate volume of the caffeine stock solution (10 mg/ml in 10 mM PBS buffer, pH 5.6) and adjusted to 1 ml final volume with PBS buffer solution. For the hydration of lipid films and for the dissolution of caffeine, the PBS buffer was used to keep the pH constant as the zeta potential depends on the pH of the sample. The zeta potential of liposomes in the absence and presence of caffeine was measured at constant lipid concentration (1 mg/ml) and varying caffeine concentration between 0 and 9 mg/ml (0 and 45 mM). The pH of the samples was always checked before the measurements, but no adjustment was required in the buffered solutions.

### EPR Spectroscopy

About 15 μl of spin-labeled MLV sample was poured into the capillary tubes and the EPR spectra were registered between 2 and 50 °C with an EMX6 Bruker X-band on-line spectrometer. For the registration 100 gauss scan range, a 2048 point resolution in a magnetic field was used. The time constant was 2.56 ms with both radicals. The spectra were registered at a microwave power of 2 and 20 mW, and modulation amplitudes of 1 and 1.5 gauss for DOX-5 and HXD-labeled samples, respectively. The sweep time was 168 and 84 s, and the conversion time was 82 and 41 ms. For the characterization of membrane fluidity, the hyperfine splitting constant was (2A_max_), read at each temperature with 2 or 5 °C intervals. 2A_max_ is the distance between the low field maximum and the high field minimum. In the case of HXD-labeled samples, the spectral simulation was applied that allows the determination of the average rotational correlation time (τ_ave_), the ordering potential (c_20_), and the order parameter (S). Details of the simulation programs and some of our earlier results obtained on liposomal systems and on proteins are given in references [20–23].

### Statistics

All the data given in the section of „Results and Discussion” were calculated as an average from at least three parallel measurements.

## Figures and Tables

**Tab. 1:** Lipid composition of lecithins from egg yolk (EL) and soybean (SL) determined from the MS spectra. Values are given in percentages. Values in brackets correspond to the figures given by the manufacturer.

chain composition	EL	SL
16:0	32 (33)	15 (14)
16:1	5 (1)	4
16:2	1	0
18:0	14 (13)	1 (4)
18:1	19 (32)	14 (11)
18:2	13 (16)	59 (64)
20:1	4	0
20:2	6	0
20:3	2	1
20:4	2 (3)	4
other	2 (5)	2 (3)
saturated	46	16
unsaturated	52	82

**Tab. 2:** pH, viscosity, and surface tension of liposomal samples prepared from soybean (SL) or egg yolk (EL) lecithin with various lipid concentrations. 0 mg/ml lecithin concentration gives the data measured for distilled water. The number of parallel charges is at least three; data are given as mean ± S.D.; n.m. denotes „not measured”

Lecithin concentr. (mg/ml)	pH		Viscosity (mPa·s)		Surface tension (mN/m)	

	SL	EL	SL	EL	SL	EL
0	5.82±0.11		0.89 ± 0.01		73.0	
0.5	6.55±0.05	5.20±0.09	0.92 ± 0.01	0.92 ± 0.01	67.1 ± 0.5	68.0 ± 0.4
2.0	6.83±0.07	4.47±0.04	0.97 ± 0.01	0.96 ± 0.01	65.2 ± 0.4	65.9 ± 0.5
5.0	6.93±0.02	4.26±0.03	1.10 ± 0.01	1.11 ± 0.01	60.5 ± 0.3	60.4 ± 0.4
7.0	7.12±0.05	4.15±0.03	1.17 ± 0.01	1.18 ± 0.01	53.0 ± 0.3	53.0 ± 0.3
10.0	7.23±0.08	4.03±0.02	1.26 ± 0.02	1.25 ± 0.01	44.3 ± 0.2	44.7 ± 0.3
12.0	n.m.	n.m.	1.31 ± 0.01	1.32 ± 0.02	41.6 ± 0.3	41.2 ± 0.2
15.0	n.m.	n.m.	1.34 ± 0.02	1.34 ± 0.02	39.6 ± 0.1	39.5 ± 0.2

**Tab. 3:** Parameters determined on the basis of non-linear least square fit to the experimental EPR spectra. Values given in the table correspond to the representative EPR spectra given in Figure 2. For the control (cont) and caffeine-containing (caff) liposome samples.

T (°C)	liposome	c_20_*	S*	τ_ave_*
2	cont	2.37	0.51	35.4
	caff	2.59	0.55	30.1

25	cont	1.76	0.39	8.1
	caff	1.76	0.39	7.4

35	cont	1.58	0.35	6.2
	caff	1.58	0.35	5.8

45	cont	1.57	0.35	3.8
	caff	1.55	0.34	3.4

* average SEM values are for c_20_: ± 0.06, for S: ± 0.02; for the average rotational correlation time a relative SEM value is the best characteristics, which is ten percentages of the best fit parameter value.
